# Quantifying Concentration Polarization – Raman Microspectroscopy for *In-Situ* Measurement in a Flat Sheet Cross-flow Nanofiltration Membrane Unit

**DOI:** 10.1038/s41598-019-52369-1

**Published:** 2019-11-04

**Authors:** Oliver Jung, Florencia Saravia, Michael Wagner, Stefan Heißler, Harald Horn

**Affiliations:** 1 Water Chemistry and Water Technology, Engler-Bunte-Institut (EBI), Karlsruhe Institute of Technology (KIT), Engler-Bunte-Ring 9, 76131 Karlsruhe, Germany; 2 DVGW Research Laboratories for Water Chemistry and Water Technology, Engler-Bunte-Ring 9, 76131 Karlsruhe, Germany; 3 Institute for Biological Interfaces 1 (IBG-1), Institute for Biological Interfaces (IBG), Karlsruhe Institute of Technology (KIT), Hermann-von-Helmholtz-Platz 1, 76344 Eggenstein-Leopoldshafen, Germany; 4 Institute of Functional Interfaces (IFG), Karlsruhe Institute of Technology (KIT), Hermann-von-Helmholtz-Platz 1, 76344 Eggenstein-Leopoldshafen, Germany

**Keywords:** Environmental sciences, Chemical engineering

## Abstract

In this work, the concentration polarization layer (CPL) of sulphate in a cross-flow membrane system was measured *in-situ* using Raman microspectroscopy (RM). The focus of this work is to introduce RM as a new tool for the study of mass transfer inside membrane channels in reverse osmosis (RO) and nanofiltration (NF) generally. Specifically, this work demonstrates how to use RM for locally resolved measurement of sulphate concentration in a cross-flow flat-sheet NF membrane flow cell with channel dimensions similar to commonly applied RO/NF spiral wound modules (channel height about 0.7 mm). Concentration polarization profiles of an aqueous magnesium sulphate solution of 10 g_sulphate_·L^−1^ were obtained at operating pressure of 10 bar and cross-flow velocities of 0.04 and 0.2 m·s^−1^. The ability of RM to provide accurate concentration profiles is discussed thoroughly. Optical effects due to refraction present one of the main challenges of the method by substantially affecting signal intensity and depth resolution. The concentration profiles obtained in this concept study are consistent with theory and show reduced CPL thickness and membrane wall concentration with increasing cross-flow velocity. The severity of CP was quantified to reach almost double the bulk concentration at the lower velocity.

## Introduction

The occurrence of concentration polarization (CP) in reverse osmosis (RO) and nanofiltration (NF) is the main underlying principle responsible for fouling phenomena such as scaling^1–3^. During cross-flow membrane operation the feed bulk solution is concentrated along the feed channel as permeate flux is induced. CP increases solute concentration across the feed channel as rejected solute builds up at and near the membrane surface. Both mechanisms superimpose, which means that the concentration polarization layer (CPL), generally, also builds up along the feed channel. Thus, in most practical applications CP is more severe closer to the outlet of a membrane module and at later stages of a multi-stage NF/RO system. Ultimately, the local increase in concentration can lead to super saturation of sparingly soluble salts, which precedes nucleation and causes the subsequent formation of a scaling layer on the membrane^4,5^. The shape of the CPL, i.e. the concentration gradient and the CPL thickness, is a function of the advective mass transport to the membrane and the advective and diffusive mass transport back to the bulk. The resultant gradient follows an exponential function^6–8^. Given no change in the operational and physical parameters and assuming no nucleation or no super saturation, CP is stable and equilibrium between the two governing mass flows is achieved quickly. The extent of CP, i.e. how much higher the concentration of a solute is at the membrane wall (c_m_) compared to the bulk concentration (c_b_), known as the concentration polarization factor (CPF) or the CP modulus (c_m_·c_b_^−1^), is dependent on many design parameters. Feed spacers increase the advective mass transport and therefore reduce the CPL. As CP generally builds up along the membrane, the length of the feed membrane channel impacts maximum solute concentration. Increased velocity reduces CP by improved mass transfer and decreased yield. Transmembrane pressure (TMP) and membrane characteristics, e.g. permeability and rejection, largely influence the CPF (also locally). Thus, the CPF is specific to a system, operating conditions and water type.

Most work on CP in NF and RO has been done on a theoretical level, modelling the phenomenon to extract important parameters such as membrane wall concentration, critical flux, CPL thickness etc. Although modelling has produced valuable results, the nature of the water, solute and membrane interactions are very complex. Consequently, current models have to either disregard or make assumptions about individual aspects of these interactions^8^. Experimental studies providing local solute concentration profiles in membrane channels are very useful for validation of modelling results. However, such studies are scarce in literature. Only few experimental studies have been presented for quantification of the CPL^9–12^. Even fewer studies have tried to measure CP in cross-flow conditions and the authors are not aware of a study presenting an experimental setup to quantify the CPL locally in membrane units with general feed channel dimensions and flow velocities present in spiral wound modules^9–11^.

In a review on CP published in 2001, Sablani *et al*. mention NMR imaging to determine CPL thickness of an oil-water emulsion in cross-flow microfiltration and a laser-based refractometric technique to measure the CPL of a biopolymer solution in dead-end ultrafiltration^9^. Since then, Fernández-Sempere *et al*. used Digital Holographic Interferometry, a variation of common Holographic Interferometry, to measure the CPL of a sodium sulphate solution in cross-flow RO^11^. The technique enables the study of concentration boundary layers by visualizing local changes in the refractive index of the sample solution. The scarcity of available experimental techniques represent the difficulty associated with localized *in-situ* study of CP on a micro-scale. Raman microspectroscopy (RM) is an additional tool available for studying concentration boundary layers *in-situ*^13^. RM is a particular promising technique as it is well established, easy to operate, has great theoretical depth resolution and sensitivity, as well as low interference with water and some common water components (e.g. NaCl)^14^. The present work introduces RM to measure, for the first time, the CPL of sulphate in cross-flow nanofiltration in a feed channel representative of spiral-wound modules.

## Theoretical Background

RM is best known as a tool for material characterization of any kind. Modern research fields include using RM for the characterization of food and water contaminants, microplastics, microorganisms and biofilms etc.^15–18^. Additionally, RM can also be used to measure concentrations of Raman active compounds in aqueous solutions, e.g. sulphate in brackish water^14^. When a sample containing Raman active compounds is exposed to a monochromatic beam of light of a certain wavelength, a portion of the incoming light is deflected from its original direction of propagation (scattered). Most of the scattered light has the same wavelength as the illumination source (Rayleigh scattering/elastic scattering). However, a small portion of the scattered light is of discretely altered wavelength, i.e. light with a significant change in frequency. This shift in wavelength corresponds to a transition in the rotational or vibrational energy state of a molecular system^19^. This phenomenon is called the Raman Effect (i.e. Raman scattering/inelastic scattering). Molecules, which exhibit this effect are considered Raman active. The Raman Effect can be used to identify and quantitatively analyse molecules in liquid phases such as water. Combining Raman spectroscopy with a confocal microscope allows for 2D and 3D quantitative analysis of the distribution of Raman active molecular systems in transparent solutions.

RM has a few important characteristics to be aware of^20–23^. First, Raman spectroscopy mostly uses a monochromatic light source in (or close to) the visible spectrum of light. As the spectral transmittance of water is high in the visible range, Raman is well suited for measurements in a water phase. Second, the Raman Effect is a very weak effect with only a very small portion of the incoming light being Raman scattered. This means that a powerful illumination source is required. Third, according to Beer’s law, absorbance is proportional to the concentration of the absorbent. Raman spectroscopy, however, relies on light scattering where such proportionality is not the case. The implication is that spectral intensities also depend on the instrument used to measure. Calibrations cannot readily be transferred to another instrument and have to be done with each instrument independently or adjusted^20^.

An important question for any type of depth profiling is that of the depth resolution. According to Juang *et al*. the minimum depth resolution can be estimated to be as follows^22^:

1 $$\Delta z\,\ge \,\pm \frac{4.4n\lambda }{2{{\rm{\pi }}(\mathrm{NA})}^{2}}$$

**Figure 1:**
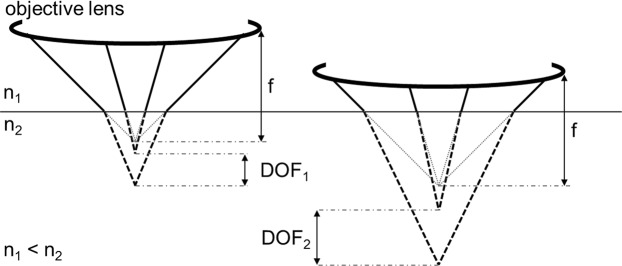
Schematic illustration of the illumination pathway when focusing into a sample with refractive index n_2_. Refraction causes a wider depth of field (DOF) and the point of focus is shifted below the nominal focal plane determined by the focal length (f) of the objective. Dashed lines resemble the path of light in the sample with n_2_. n: refractive index; Note: The setup used in this work actually exhibits two interfaces with changes in refractive index, i.e. air to flow cell window and flow cell window to aqueous solution. Since the objective lens is corrected for the flow cell window (cover glass correction of 1.3 mm) and for simplicity reasons, the windows’ influence on the light path is disregarded.

Thus, the depth resolution depends on the refraction index of the immersion medium *n*, the wavelength of the illuminating light *λ* and the numerical aperture (NA) of the objective lens. For the RM setup used in this work (water: n = 1.33, lens: NA = 0.7, laser: *λ* = 532 nm), the minimal depth resolution would be as small as 2 µm. However, as Everall has pointed out, the depth resolution can be substantially worse when the optical beam is refracted due to the occurrence of spherical aberration^24,25^. Figure 1 demonstrates what happens to the optical pathway when there is an interface at which the refraction index increases, e.g. air to water. Due to refraction, the focus point is shifted below the nominal focal plane, which would otherwise be determined by the focal length, *f*, of the objective. In depth profiling this causes a foreshortened representation of the actual depth profile and an underestimation of the thickness of the sampled volume. Additionally, the spherical aberration also causes an increasing depth of field, *DOF*, the deeper the focus into the sample. This means that depth resolution degrades when focusing deep into the sample. The use of a confocal aperture can restore some of the lost depth resolution although accompanied by major loss of signal intensity as signal originating from outside the focal plane is clipped at the confocal aperture^26^. Finally, laser intensity too is decreasing with depth as spherical aberration causes a broadening of the illumination volume. In total, spherical aberration alters the expected depth profile substantially, which has important consequences to the interpretation of the acquired depth profile data as well as to the experimental methodology and setup required.

## Materials and Methods

### Experimental setup

A membrane flow cell has been designed specifically for this work to allow for the simulation of practical conditions in common spiral wound modules in combination with usability for RM. The flow cell is a flat sheet cross-flow membrane unit with a channel length of 11.2 cm and channel width of 3 cm for a total membrane area of 33.6 cm². Thickness of the feed channel is about 700 µm. The flow cell features two sapphire windows of 1.3 mm in thickness to permit 3D Raman sampling while maintaining cell integrity at higher pressures. The cell has been operated successfully at pressures up to 12 bar with the featured window thickness of 1.3 mm. A preliminary test with a sapphire window of smaller size and a thickness of 1 mm has shown structural integrity at 40 bar, demonstrating the principal applicability of this cell design for the simulation of common RO and NF applications. Window thickness is an important parameter as it increases the required working distance of the objective as well as spherical aberration, which both negatively affect depth resolution.

**Figure 2:**
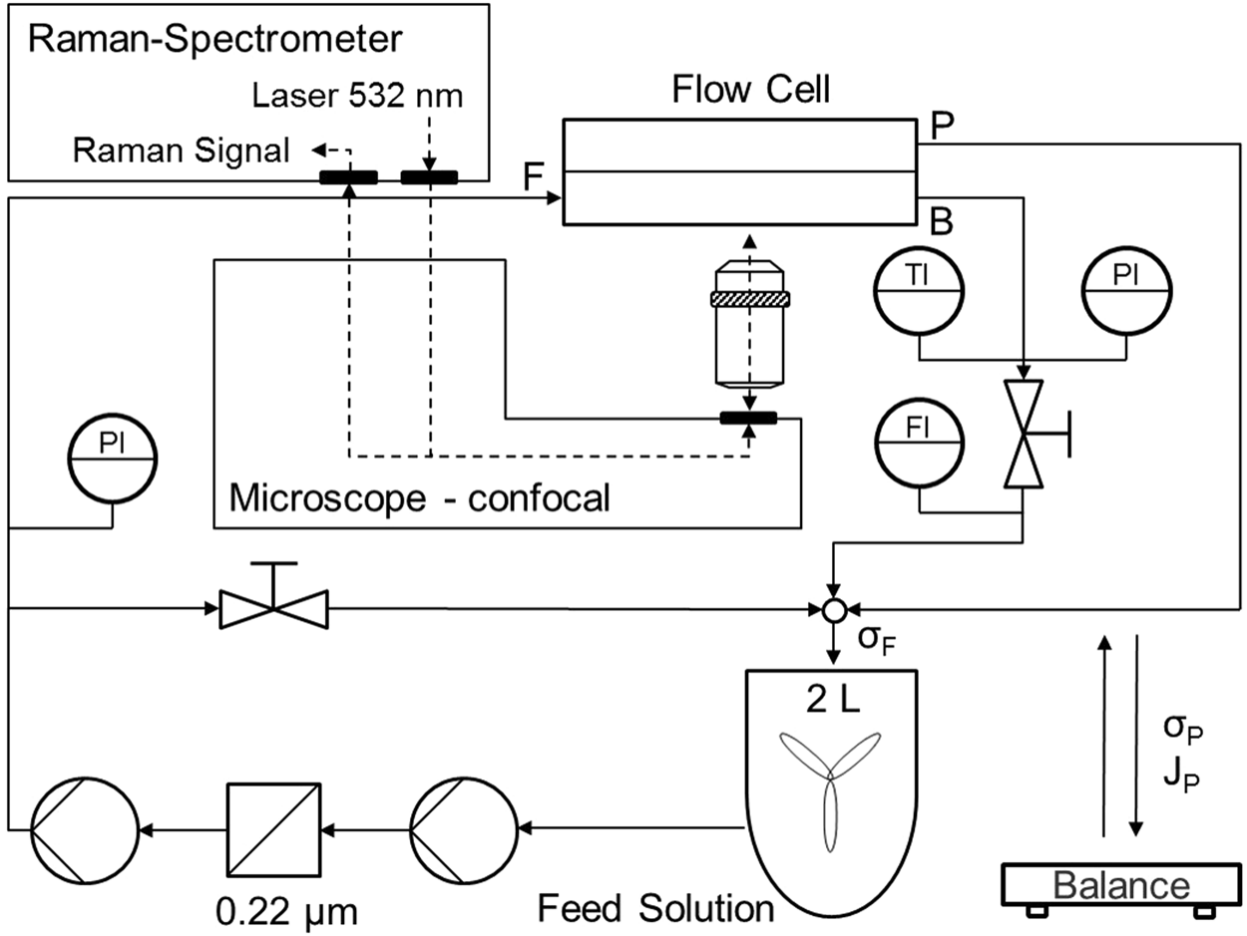
Scheme of the experimental setup combining nanofiltration in recirculation mode and Raman microspectroscopy. The microscope is of inverted configuration. The membrane is positioned on top of the feed channel. Flow, pressure, conductivity, flux and temperature are recorded and kept constant during measurements. The feed solution is a pure magnesium sulphate solution, which is well below saturation. No scaling occurs. The permeate flux is measured by weighing. F: Feed, B: Brine, P: Permeate, σ: electrical conductivity, J: Flux.

The membrane filtration system is a total recirculation system set up to keep all parameters constant. Figure 2 shows a scheme of the principal setup. Note that the configuration of the microscope is inverted, which means that the membrane is located on top of the feed channel. The sample volume is a 2 L container, which is continuously stirred and temperature regulated. The feed solution is pumped through a 0.22 µm particle filter, which is followed by a high pressure pump. A recirculation bypass including a metering valve is used to regulate feed flow. Permeate is re-joined with the brine behind the pressure valve and then routed back into the feed container. This is done jointly with the recirculation flow. Permeate can also be routed across a balance to determine permeate flux and permeate conductivity. This was not done during Raman measurement operation but rather before and after the start of a measurement series. Measurement parameters were recorded using NI LabVIEW™. Recorded parameters were temperature, feed and permeate conductivity, brine and permeate flow as well as inlet and outlet pressure.

Feed solution was a magnesium sulphate solution of varying concentrations from 7 g·L^−1^ (0.07 mol·kg^−1^) up to 33.5 g·L^−1^ sulphate (0.35 mol·kg^−1^). The solution was prepared with MgSO_4_·7H_2_O from Roth (99.7% purity) and deionized water. Sulphate is a common component of scaling in the form of gypsum. The solubility of magnesium sulphate is 300 g·L^−1^, thus precluding the occurrence of scaling in these experiments. Without membrane fouling and with all parameters constant, the CPL is stable after reaching equilibrium conditions and the measurement is not time sensitive. Measurements of the CPL were done with a solution of 10 g·L^−1^ sulphate (0.104 mol·kg^−1^) at multiple cross-flow velocities and with constant operating pressure of 10 bar.

The pressure difference across the membrane is the driving force for reverse osmosis driven membrane processes. However, the effective transmembrane pressure (TMP) differs from operating pressure due to the osmotic pressure (II) of the feed and flux is induced only when effective TMP exceeds the osmotic pressure of the feed solution. Therefore, system pressure has to be higher than the osmotic pressure of the feed water. Since in the CPL osmotic pressure increases locally towards the membrane wall, CP reduces effective TMP. Consequently, the extent of CP is also limited by the applied operating pressure since effective TMP must be greater zero to allow CP formation in the first place. The osmotic pressure can be estimated using the Van’t Hoff equation

2 $$\Pi =i{\rm{\varphi }}{\rm{mRT}}$$

with *i* being the number of dissociation of the salt, *m* being molality, R being the gas constant, T being the temperature in K and *ϕ* being the osmotic coefficient (i.e. NaCl: ϕ = 0.925 at 0.2 mol·kg^−1^ ^27^; MgSO_4_: ϕ = 0.556 at 0.2 mol·kg^−1^)^28^.

The relationship between sulphate concentration and osmotic pressure of a pure magnesium sulphate solution in the present range of concentration is thus given by

3 $$\Pi =0.091\cdot {\rm{m}}\cdot {\rm{T}}$$

with II in bar, molality in mol·kg^−1^, temperature in K. For the CPL measurement conditions (0.104 mol·kg^−1^) the effective TMP at 20 °C, thus, is 7.2 bar initially, before the formation of the CPL. With CPL formation the effective TMP reduces. Osmotic pressure of the feed solution is equal to the applied pressure of 10 bar at a concentration of about 35.5 g·L^−1^ (0.37 mol·kg^−1^). This value provides an upper reference for the calibration requirements.

The Raman system used was an inverted Raman microscope SENTERRA I from Bruker. Recording software was OPUS 7. A 532 nm laser (Cobolt Lasers, Solna, Sweden) with a power of 50 mW was used for all measurements. The objective, Olympus LUCPLFLN 60 × , has a NA of 0.7, working distance of 1.5 mm beyond the cover glass, correction collar for a cover glass thickness of up to 1.3 mm and a magnification factor of 60 × . The membrane cell was mounted onto the sampling stage of the Raman microscope. The cell windows cover two areas accessible for analysis. One area in the beginning of the flow channel, 1.5 to 3.5 cm from the inlet and another area 7 to 9 cm from the inlet. The results presented in this work were all measured on a fixed position in the middle of the feed channel, 8.5 cm from the inlet.

Sulphate has nine modes of internal vibration that are Raman active of which the linear symmetrical stretching vibrational mode (*ν*_1_) is the strongest. It shows a Raman band with a peak at 981 cm^−1^. The intensity of the Raman band (integral area 994-966 cm^−1^) is proportional to the concentration of sulphate molecules in the focus point. This work excludes other ions, mainly sodium chloride, in the sample solution in order to keep osmotic pressure low and increase flux. However, Murata *et al*. have shown that the linear correlation of Raman signal to concentration is not influenced by the presence of sodium chloride up to a concentration of 58 g·L^−1^ ^14^. The applicability of the presented method should thus be extendible for particle free natural salt waters.

All filtration experiments were done with a DOW FILMTEC™ NF270 nanofiltration membrane. The NF270 has a nominal rejection of magnesium sulphate of >97% and a permeability of 11.1 L·m^−2^·h^−1^·bar^−1^ according to the manufacturers specifications. Clean water flux in the filtration cell at 10 bar pressure was 7.36 mL·min^−1^ (Permeability 13.1 L·m^−2^·h^−1^·bar^−1^). Rejection of magnesium sulphate solution of 10 g·L^−1^ sulphate was 97.6% in terms of conductivity. The NF270 was chosen for these experiments for its high permeability, high rejection for sulphate, lack of interfering Raman bands in the range of 994-966 cm^−1^ (sulphate band area *v*_1_), lack of fluorescence and widespread commercial use. The NF270 is a polyamide thin-film composite membrane with a supporting layer made of PES, which shows three distinct Raman bands in the range of 1165-1060 cm^−1^. A raw spectra showing the Raman bands of the membrane and the Raman band *v*_1_ of sulphate is given in supplementary information (SI) Fig. S1. The intensity of these Raman bands is later referred to as the “membrane signal” and the “sulphate signal” respectively.

### Experimental methodology

All relevant parameters were kept constant during the recording of the CPL profiles. Consecutive measurements assure steady state was achieved. Feed concentration was set measuring the electrical conductivity at 25 °C with a conductivity of 9.55 mS·cm^−1^ corresponding to a concentration of 10 g·L^−1^ sulphate. Feed pressure was held constant at 10 bar and feed temperature at 21 °C. Depth profiles were recorded for velocities of 0.04 m·s^−1^ and 0.2 m·s^−1^.

The raw data depicts the Raman intensity over *z* (distance from the membrane) and requires a conversion to display the CP profile. For the conversion a calibration was set up to correlate the Raman intensity to the sulphate concentration. Calibration was done with a velocity of 0.2 m·s^−1^. Feed pressure was about 0.15 bar, which was the minimum pressure required to set the desired velocity. Depth profiles of seven concentrations, 7, 10, 15, 20, 25, 30 and 33.5 g·L^−1^ sulphate, were recorded for one calibration data set. In total four data sets were recorded and averaged. A linear fit across all concentrations for each point of depth was used to give the correlation of sulphate concentration to Raman intensity dependent on the position of the focal plane in relation to the membrane. The calibration data set is included in SI Fig. S2. The linear fitting functions for each depth point are listed in SI Table S3.

Depth profiles were recorded with a step width of 10 µm and a range of 250 µm. The recorded spectra yield the sulphate signal and the membrane signal simultaneously. The point at which the membrane signal reaches maximum value was set to *z* = 0 µm (set location of the membrane surface). Presented are the measurement values in the range −20 to 170 µm. The measurement parameters for the Raman system were the same for all recordings presented. The total exposure time was split in consecutive five second intervals of exposure (integration time t_i_) per measurement position. The software gives a joint output (co-edition) of one spectra after the total exposure time of 30 seconds (integration time t_i _=5 s, co-edition = 6). Thus, the total measurement time of a depth scan with 25 points is about 14 minutes (including initializing of the Raman spectrograph and background recording). The nominal laser intensity was set to 50 mW power. A background was measured before each measurement. The confocal aperture was set to a 50 × 1000  µm slit. Although a smaller pinhole aperture (25 µm) was available and would suggest improvements in depth resolution, it was decided against in order to compromise with measurement time. The bigger slit aperture causes much less intensity loss, which allowed for a 20 times shorter integration time without substantially reducing depth resolution. This is further discussed in the following section.

## Results and Discussion

Measuring CP with RM is not a straight forward technique. After data collection, the Raman intensity needs to be converted into concentration. Due to complex optical effects, which need to be accounted for, the chosen method for data conversion has a large influence on the final shape and quantification of the CPL. The better the conversion method corrects for the optical distortions, the more accurate the plot of the CPL will be. Thus, three steps are necessary to yield accurate results. Firstly, the relationship of Raman intensity with concentration needs to be established. Secondly, the influence of optical distortions on the Raman intensity distribution through the feed channel (depth profile) needs to be discussed and thirdly, the effect of the optical distortions on the chosen conversion method and on the final CPL profile has to be examined.

### Raman intensity distribution vs. sulphate concentration

**Figure 3:**
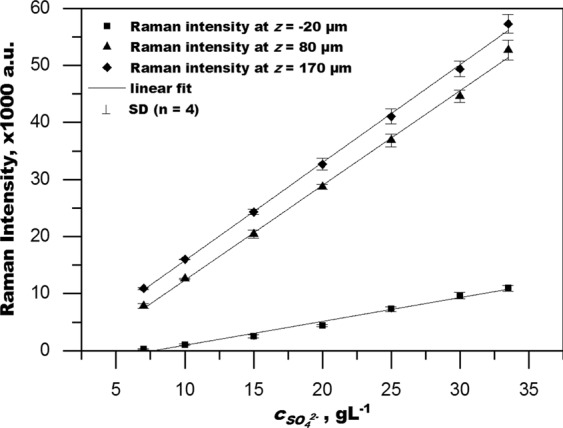
Calibration results. A full calibration (7–33.5 g_sulphate_L^−1^) was performed for every point along the depth profile from z = −20 µm to z = 170 µm with Δz = 10 µm (z = 0 µm being the position of the membrane surface). For illustration purpose only calibration curves for depth points z = −20, 80 and 170 µm are presented. Complete calibration results are summarized in SI Table S3. SD: standard deviation. R^2^ > 0.978 for all curves.

Raman spectroscopy provides a spectrum of Raman intensity counts over wavenumber shift. The integral of the Raman band at 981 cm^−1^ (integral area 994-965 cm^−1^) emanating from sulphate is proportional to the sulphate concentration. This is shown in Fig. 3 for three positions *z* = −20, 80 and 170 µm (membrane surface at z = 0 µm, positive values refer to a position inside the feed channel away from the membrane). Similar correlations were done for each point of the depth scale, which are included in SI Table S3. Indeed, the correlation has to be established for each point of the depth profile individually, since the signal is losing in intensity and the slope is decreasing when focusing deeper into the sample. This is caused by the present refraction interface as shown in Fig. 1. To discuss this further, we have to look at how the output data is affected by the spherical aberration.

**Figure 4:**
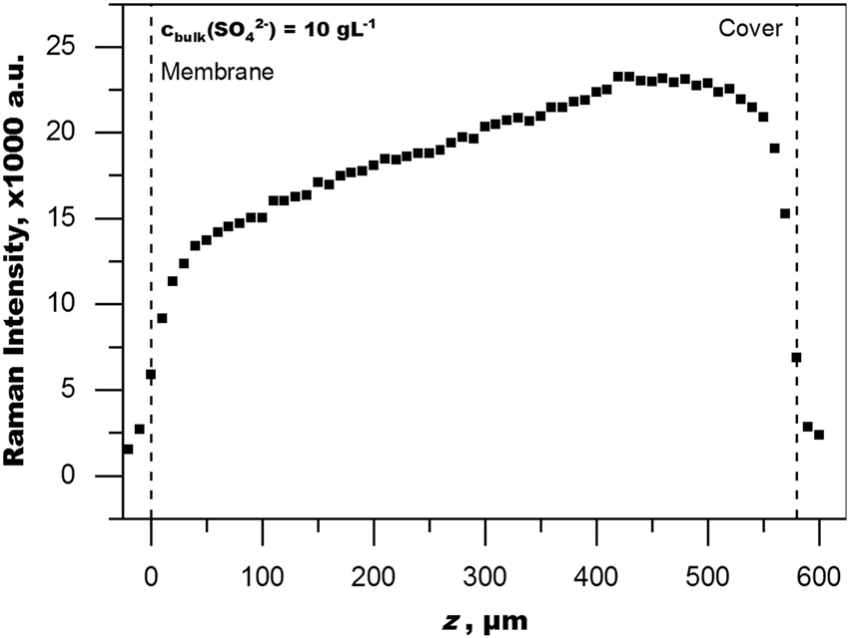
Depth profile through the feed channel of a homogenous magnesium sulphate solution of c(SO_4_^2−^) = 10 g·L^−1^. Plotted is the raw data of the sulphate band area (994-965 cm^−1^). The inhomogeneous intensity distribution is caused by spherical aberration due to refraction. This also causes foreshortening of the profile and thus an inaccurate representation of the thickness of the feed channel which is about 700 µm.

The influence of the optical effects on the Raman intensity distribution through the feed channel can be illustrated by plotting the raw data of a depth profile through the whole feed channel with an unpressurized magnesium sulphate solution as shown in Fig. 4. Although the sulphate concentration is constant throughout the feed channel, the Raman intensity is continuously decreasing towards the membrane. The Raman intensity distribution can be explained by (1) decreasing laser intensity (i.e. power density: mW·mm^−2^) with deeper penetration into the sample^26^. The laser intensity decrease is linear and correlates well with the linear decrease of Raman intensity through most of the feed channel. The clipping of the Raman intensity near the borders of the feed channel is caused by (2) overlap of the focal volume (effective illumination volume) with the feed solution and the membrane respectively the cover. The cover (sapphire) and the membrane do not contain relevant concentrations of sulphate and thus do not contribute to signal intensity. The overlap is starting where the signal decrease deviates from linearity. For these reasons, the linear correlation between Raman intensity and sulphate concentration is dependent on the penetration depth. It should also be noted that the feed channel thickness is not represented accurately in Fig. 4. This is due to spherical aberration, which causes a foreshortened representation of the feed channel depth as demonstrated in Fig. 1.

The clipping of the Raman intensity of sulphate due to the cover is not a sharp cut-off. This is because the focal volume (i.e. DOF) is not a sharp point of focus but rather an intensity distribution. The laser beam is focused through the objective into the sample onto the focal plane. Due to the wave characteristics of light, constructive and destructive interference lead to a pattern with its highest intensity at the focal plane and areas with diminishing intensity to either side of the focal plane. Thus, excitation of Raman active species is not limited to the focal plane but has diminishing contributions from above and underneath the focal plane. The same happens in return, where the scattered light originating at the focal point creates a similar interference pattern at the spectrograph. This particular interference pattern is called a point spread function (PSF).

**Figure 5:**
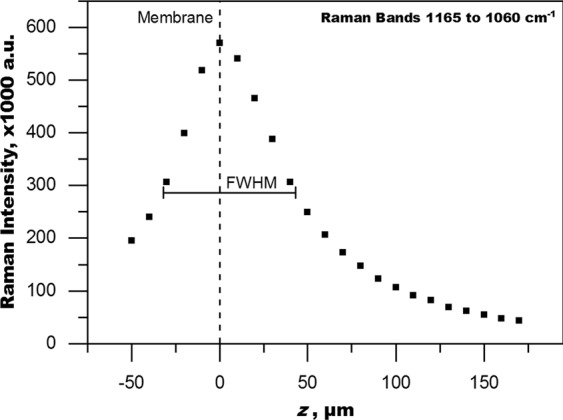
Raman intensity distribution of the membrane bands at 1165-1060 cm^−1^ over z (optical axis). The plot represents the PSF (point spread function) with FWHM = 75 µm (full width at half maximum). The maximum intensity is used as the reference for the position of the membrane surface.

It is helpful to consider the Raman data from the membrane to understand the extent of blurring present with this particular setup. Figure 5 shows the plot of the Raman intensity of the membrane bands (1165-1060 cm^−1^) over *z*. The almost symmetrical shape of the plot is a reasonable representation of the PSF of the present setup. All of the membrane signal originates from a plane at *z* = 0 µm, which is the location of the membrane surface. However, the signal is present (with decreasing intensity) even when focusing away from the membrane surface. The PSF characterizes this distribution.

A PSF is commonly categorized by the full width at half maximum (FWHM), which is the width of the function at half the maximum intensity. The FWHM is a representation of the depth resolution. In an ideal setup, the PSF would be sharp and symmetrical with a maximum depth resolution of 2 µm for the present setup in a dry case scenario (Eq. 1). However, due to the refraction in the water phase, the actual PSF is wider, asymmetrical and broadens further the deeper the focus plane. From the plot of the membrane signal in Fig. 5 the FWHM of the present setup can be estimated to be about 75 µm. This shows the extent of the influence of spherical aberration due to refraction at the water interface. Any means to mitigate or account for this effect will substantially improve the measurement technique in terms of depth resolution. The confocal aperture also influences the width of the PSF. A smaller pinhole increases depth resolution by clipping light, which originates from outside the focal plane. However, our measurements with the 25 µm pinhole aperture yield a depth resolution of about 65 µm. This is an improvement of roughly 10 to 15% but the loss in intensity is substantial. As a result, measuring time increases about 20-fold to make up for the low intensity, while the depth resolution remains relatively poor. Improvements in depth resolution of 50% or better would be desirable.

**Figure 6:**
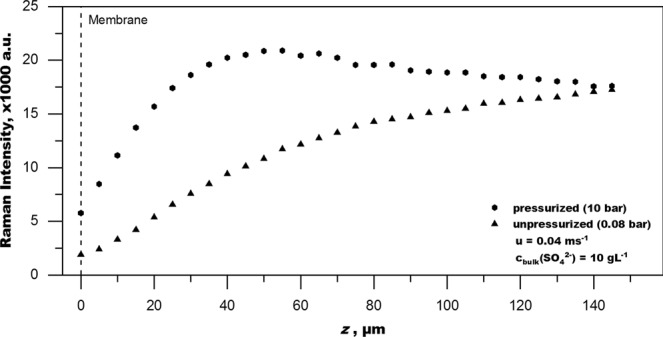
Raman intensity distribution of sulphate (981 cm^−1^) in the feed channel near the membrane (z = 0 µm) with (pressurized) and without (unpressurized) the presence of concentration polarization (CP). Data acquired at a mean velocity u = 0.04 m·s^−1^. Sulphate concentration in the fully mixed bulk was 10 g·L^−1^.

A broad PSF means that there is a lot of contribution to the Raman signal intensity from outside the focal plane. This is important to consider when interpreting measurement data. However, the raw signal profiles (Figs 4–6) also demonstrate that the Raman measurement is sensitive enough to sufficiently resolve changes in Raman intensity with a resolution smaller than 5 µm. It is thus fair to assume that concentration changes can be recorded similarly and with similar resolution if one accounts for the effect of diminishing Raman intensity with depth.

### Concentration polarization & calibration as method for conversion

The capability of RM to show the CPL is demonstrated well by plotting the raw data of the Raman intensity of the sulphate band of pressurized operation (induced CP) versus the raw data of unpressurized operation (no CP), which is shown in Fig. 6. The two depth profiles are clearly distinct and sufficiently resolved. Both depth profiles have been recorded with the same velocity and the same bulk solution. The depth profile of the unpressurized system is constantly decreasing as explained in the previous section. The depth profile of pressurized operation shows an increase in Raman intensity with a maximum closer to the membrane. This increase in Raman intensity can only be caused by an increase in sulphate concentration. The shape is the result of superposition of (1) Raman intensity increase due to increased sulphate concentration towards the membrane and (2) diminishing Raman intensity due to overlap of the PSF with the membrane and diminishing laser intensity with depth, which is independent of the mode of operation.

In order to extract a sulphate concentration profile from the Raman intensity data, data processing needs to account for the optical distortions and the blurriness of the focus point. One option to do this conversion is by calibration. The necessary assumption is that the optical effects of spherical aberration are identical with and without the occurrence of CP. This assumption is justified, when the optical pathway remains the same in both cases, which means that there are no changes in the refractive index. Indeed, the change in refractive index expected from the highest concentration in the CPL to the bulk concentration is only about 0.15% total^29^.

If the influence of changes in refractive index are neglected, then the shape of the PSF are also identical in both modes of operation. This means that the overlay of the PSF with the membrane is the same for both modes with reference to the membrane position, which in turn is fixed to the position of the maximum of the Raman membrane signal. This methodology automatically accounts for the compression of the membrane, which occurs in pressurized operation. For the present setup, compression of the NF270 membrane at 10 bar operational pressure is only about 5 to 10 µm. In pressurized operation, the focus point is shifted deeper into the sample by that amount. This effect can be influential when compression is more severe.

Assuming the PSF identical regardless of sulphate concentration, a practical calibration is possible, which corrects for the loss of Raman intensity due to spherical aberration. However, the calibration has to be done for each individual point of the depth scale, i.e. the calibration data set must be recorded as a depth profile as well. Multiple profiles at varying sulphate concentration in fully mixed conditions then permit to relate the Raman intensity measured during the CPL measurement to sulphate concentration. Fully mixed conditions can be assumed when no flux occurs during cross-flow operation (unpressurized operation). Examples for the linear correlation of Raman intensity to sulphate concentration for three points of the depth scale were shown in Fig. 3.

**Figure 7:**
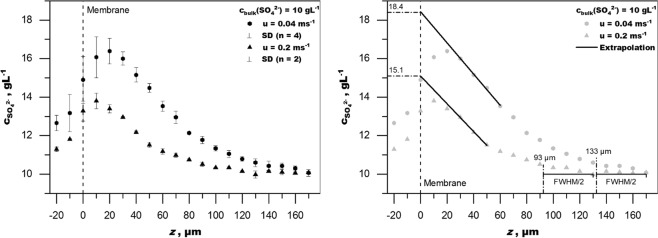
Left: CP profiles for a magnesium sulphate solution of 10 g·L^−1^ sulphate at p = 10 bar and velocities u = 0.04 m·s^−1^ and u = 0.2 m·s^−1^. SD: standard deviation Right: Linear extrapolation to the membrane surface to roughly estimate membrane wall concentration and true thickness of CPL after correction with FWHM/2.

**Figure 8:**
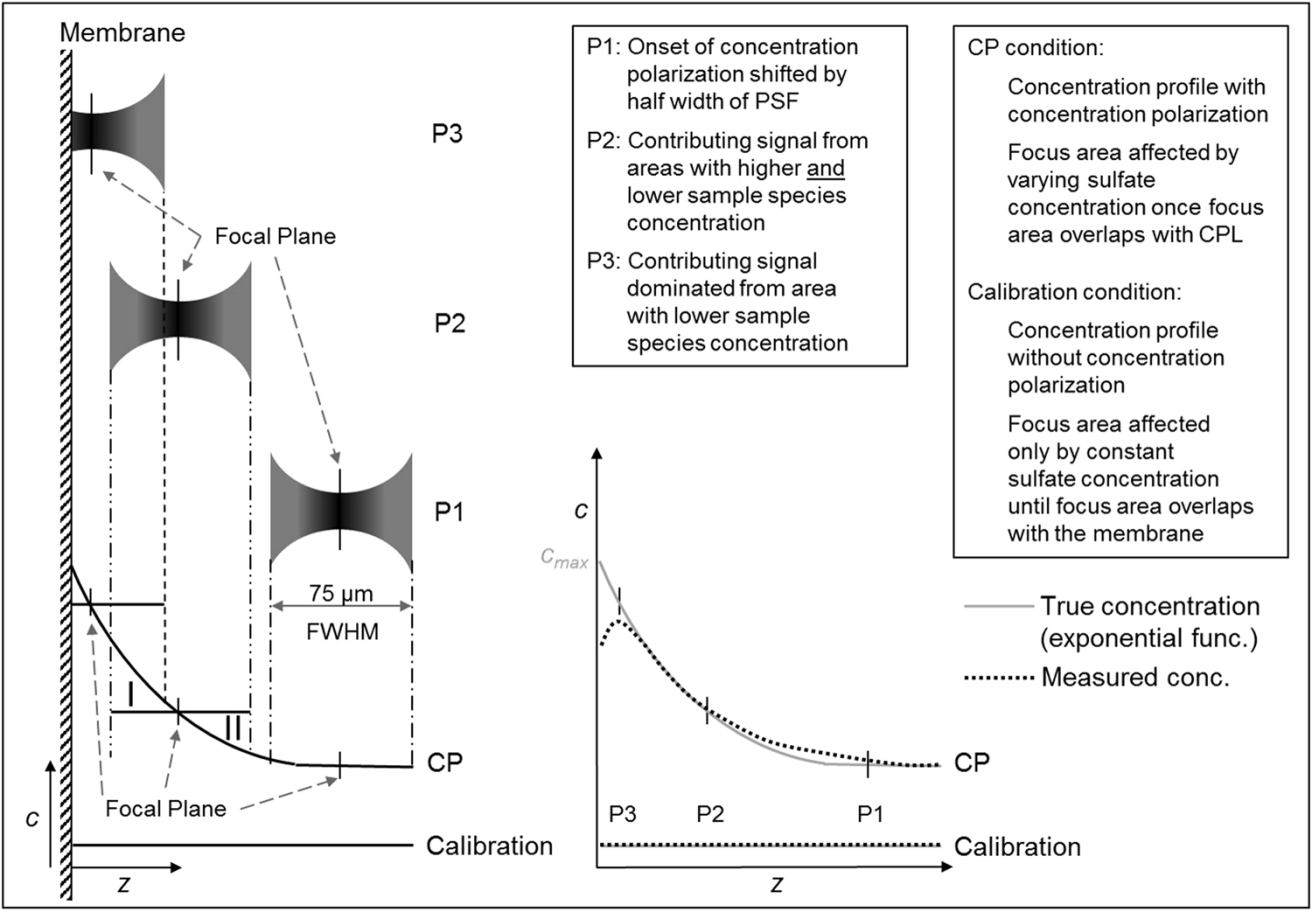
Influence of the PSF on the shape of the sulphate concentration depth profiles shown in Fig. 7.

The conversion of the Raman intensity profiles of pressurized operation result in the CP profiles depicted in Fig. 7. The CP depths profiles show a gradual increase in concentration with an exponential shape, as would be expected from theory, until *z* = 20 µm. Closer 20 µm, the data points show a decrease in concentration. This is a result of the methodology and experimental setup. As previously demonstrated with the evaluation of the membrane signal in Fig. 5, the focus point is substantially blurred and there is overlap with the opaque membrane. For comprehensive understanding the following issues have to be considered when interpreting the profiles, which are all related to the depth resolution. (1) The onset of the CPL (i.e. CPL thickness), (2) the value at the membrane (membrane wall concentration, c_m_) which is also c_max_, and (3) the plausibility of the concentration values. First, the value of CPL thickness can be taken from the graph only with the width of the PSF in mind. Assuming the direction of measurement being towards the membrane, a raise in concentration will be observed before the focal plane matches the actual onset of the CPL. Figure 8 position 1 shows a graphical explanation. This shift depends on the width of the PSF. Hence, FWHM/2 can be used as a correction as shown in Fig. 7 on the right. For the setup used to record this data, the FWHM is about 75 µm. Hence, the boundary layer thickness is about 37.5 µm less than the point of first deviation from the baseline.

The shape of the CPL profile close to the membrane (*z* < 30 µm) can be explained with similar considerations. As shown in Fig. 8 position 3, the contribution to the signal from outside the focus plane originates mainly from an area with lower concentration than present at the focal plane. Whereas in the case of calibration, the concentration at the focal plane is the same as the concentration across the total width of the PSF. This results in the data points close to the membrane being undervalued. In fact, all data points closer than FWHM/2 to the membrane can be assumed to be undervalued. The increasing uncertainty (standard deviation) towards the membrane further demonstrates the challenges with measurement close to the membrane wall. The examination shows that, using this methodology, accurate data is obtained when signal contribution from outside the focus plane matches the signal intensity, which is obtained at the same depth in calibration. In other words, if the added Raman intensity contribution from underneath the focal plane (area I in Fig. 8) equals the lesser Raman intensity contribution from above the focal plane (area II in Fig. 8), then the measured value for CP equals the measured calibration value at that specific point of the depth scale. This can be seen in principle in Fig. 8 at position 2. It is reasonable to assume such symmetry in the middle (near linear) section of the profile. Hence, the middle section of the profile should yield the most accurate results.

The membrane wall concentration thus cannot, unfortunately, be conveniently extracted from the Raman intensity data using this experimental methodology. A much sharper PSF than achievable with the present setup or a model correcting for the wider PSF in other ways would be required for this task. However, since the mid-section of the CPL is represented accurately, a reasonable estimate of the membrane wall concentration can be achieved by linear extrapolation from the mid-section to *z* = 0 µm. The membrane wall concentration (c_m_) and the CPF (c_m_·c_b_^−1^; c_b_: bulk concentration) derived from it, are important parameters for flux, rejection and scaling considerations. Therefore, the estimate from linear extrapolation provides an important quantification from an *in-situ* measurement method. The membrane wall concentration could also be extrapolated by fitting an exponential function to the measured profile. This would make sense since the CPL is an exponential function in theory. However, as an exponential function is more sensitive to variations in the gradient, the extrapolated value at *z* = 0 has great uncertainty. Since the purpose of this study is to introduce, demonstrate and discuss this new technique, linear extrapolation avoids the otherwise necessary rigor in mathematical treatment. However, optical improvements reducing the FWHM (e.g. increased NA) would make fitting of an exponential function to the measurement points more feasible and more precise extrapolation of membrane wall concentration could be achieved.

Direct measurement of the membrane wall concentration could be an area of special consideration using the same principal technique. The setup can be adjusted to get close to the achievable optimum of less than 2 µm in depth resolution by using an immersion objective with a high NA. Such a setup would have a much shorter working distance but in turn, due to the immersion, would exhibit less optical distortions decreasing effective depth resolution. The challenge of such a setup would be to solve issues like the obstruction of feed channel flow by the objective, pressure resistance and sealing.

In conclusion, these considerations allow for the extraction of some important parameters from the sulphate concentration plot (Fig. 7). For a bulk sulphate concentration of 10 g·L^−1^, linear extrapolation (data points *z* = 30 to 50/60 µm) to the membrane gives a membrane wall concentration (c_m_) of sulphate of about 18 g·L^−1^ at a velocity of 0.04 m·s^−1^ (CPF = 1.8) and 15 g·L^−1^ at a velocity of 0.2 m·s^−1^ (CPF = 1.5) at 7.2 bar TMP. The boundary layer thickness is about 130 µm and 90 µm respectively. Both values, thickness and wall concentration, are subject to some uncertainty since they are derived from extrapolation and fitting to the measurement values and should be interpreted accordingly. Reproducibility of measurements is good. Standard deviation (SD) between independent measurements is about 2%, which corresponds to about 0.2 g·L^−1^ absolute for the sample solution of 10 g·L^−1^ sulphate concentration in the bulk. The SD increases in the area 0 to 20 µm, which however is inconsequential since the measurement technique fails to produce accurate values in that area due to the overlap of the PSF with the opaque membrane.

The extent of CP (i.e. the CPF) measured with this new method is within plausible range as reported in literature^7,30,31^. Salcedo-Díaz *et al*. measured CPL using Digital Holographic Interferometry in a slit-type channel with a sodium sulphate solution and about similar active membrane area, Reynolds numbers and channel length. The authors report a CPF between 1.7 and 1.2 for the low Re case and 1.4 and 1.1 for the higher Re case^31^. However, a comparison with different setups and simulations in literature is of limited value as the CPL characteristics are very dependent on the system, the operating conditions and the water type. Among the important parameters specific for the system presented herein are the use of a pure magnesium sulphate solution, which has 40% less osmotic pressure than a sodium chloride solution of the same molality, a narrow feed channel with a thickness similar to commercial spiral wound modules but without feed spacer, a channel length of only 8.5 cm before the point of measurement and an active membrane area of only 33.6 cm².

The spherical aberration occurring with the setup used in this work cannot easily be remedied. If the goal is to image CPL in practical RO membrane application, a transparent cover is necessary to enclose the pressurized feed channel and to not obstruct feed channel flow. The feed channel is also of a certain thickness, typically about 0.8 mm in common RO modules, and the working distance of the objective lens has to be long enough to cover the entire feed channel height to the membrane surface. Thus, the objective lens has to correct for the coverslip and deep penetration into refractive media while maintaining a high numerical aperture. These are extraordinary requirements for an objective lens. Mathematical modelling in order to predict depth resolution and depth scale compression is also complex^26^. The use of a confocal aperture can restore some of the loss in depth resolution but at the cost of significant Raman intensity loss. Nevertheless, the technique shows that CPL can be recorded until close range to the membrane and in practical flow conditions. Furthermore, the technique offers the possibility to also measure CPL with a spacer present in the feed membrane channel since the optical axis is in *z* and the spatial resolution in the xy-plane is high. Thus, 3D measurement of the sulphate concentration distribution inside individual spacer mesh elements is achievable.

## Conclusion

The present work demonstrates the applicability of RM for the measurement of CP in a NF setup representative of commercial spiral wound modules. The major challenge with the setup is the occurrence of spherical aberration, which causes a deterioration of the depth resolution and widening of the PSF. The theoretical minimal achievable depth resolution of about 2 µm cannot be reached with the present setup. Instead, the depth resolution near the membrane surface is only about 75 µm. Nevertheless, since CP is a continuous concentration profile, depth profiles can be recorded with a resolution of less than 5 µm by observing the changes in Raman intensity throughout the depth profile. Therefore, the main finding of this study is the difference in Raman intensity profiles between unpressurized/no-flux and pressurized/flux operation shown in Fig. 6. The difference in profiles is entirely caused by CP. Due to the linear correlation of Raman intensity and sulphate concentration, the Raman intensity data can be converted to quantify CP. However, the conversion of the raw data into concentration values is hindered by the optical distortions present. This study used a calibration approach to correct for the complex optical effects. This approach produces a viable sulphate concentration profile, which however cannot resolve data points close to the membrane surface (0 to 20 µm). A reasonable estimate of the membrane wall concentration and the CPF can be obtained by extrapolation. The thickness of the boundary layer can be corrected with FWHM/2 to account for the broader PSF. Other conversion options, in particular an approach assisted by mathematical modelling of the optical effects, should be explored to improve results.

This work used the best simple setup available as well as a simple calibration routine with no sophisticated mathematical editing. The CPL was imaged successfully at velocities of 0.04 m·s^−1^ and 0.2 m·s^−1^. The concentration polarization factor could be estimated from the profile to be about 1.8 for low velocity and 1.5 for high velocity respectively. The present setup did not use spacers nor did it utilize a natural brackish water feed in order to simplify fluid dynamics and optics as well as maximize the CPL. The setup however does not limit the applicability of spacers and Murata *et al*. have shown that Raman spectroscopy can be used for the measurement of sulphate in natural brackish waters^14^. Furthermore, since RM is a strong tool for material characterization, it allows for the differentiation of dissolved compounds (sulphate_(aq)_) and solids (e.g. crystals of gypsum). The herein presented method demonstrates on a specific example of NF with sulphate how to achieve quantitative assessment of the CPL of Raman active compounds in membrane applications such as NF and RO. Yet it can be applied more broadly to characterize mass transfer in feed membrane channels and may also be applicable to related fouling phenomena. It provides experimental *in-situ* data in a research area where such data is scarce in literature and which relies primarily on modelling.

## Supplementary information

Supplementary Information

## Data Availability

The datasets generated during and/or analysed during the current study are available from the corresponding author on reasonable request.
