# Supplementary material for: Quantifying Concentration Polarization – Raman Microspectroscopy for In-Situ Measurement in a Flat Sheet Cross-flow Nanofiltration Membrane Unit
Source: Sci Rep. 2019 Nov 4;9:15885. doi: 10.1038/s41598-019-52369-1 (PMC6828971; doi:10.1038/s41598-019-52369-1)
Supplement: Supplementary file 1 — Supplementary Information [file 41598_2019_52369_MOESM1_ESM.doc]

**Quantifying Concentration Polarization – Raman Microspectroscopy for *In-Situ* Measurement in a Flat Sheet Cross-flow Nanofiltration Membrane Unit**

*Oliver Jung^a^, Florencia Saravia^b^, Michael Wagner^c^, Stefan Heißler^d^, Harald Horn^a,b^**

**Corresponding author***: Harald Horn; harald.horn@kit.edu;* *Karlsruhe Institute of Technology (KIT), Engler-Bunte-Institut, Water Chemistry and Water Technology,* *Engler-Bunte-Ring 9, 76131 Karlsruhe, Germany*

**Affiliations**

*^a^ Water Chemistry and Water Technology, Engler-Bunte-Institut (EBI), Karlsruhe Institute of Technology (KIT), Engler-Bunte-Ring 9, 76131 Karlsruhe, Germany*

*^b^* *DVGW Research Laboratories for Water Chemistry and Water Technology, Engler-Bunte-Ring 9, 76131 Karlsruhe, Germany*

*^c^ Institute for Biological Interfaces 1 (IBG-1), Institute of Biological interfaces (IBG), Karlsruhe Institute of Technology (KIT), Hermann-von-Helmholtz-Platz 1, 76344 Eggenstein-Leopoldshafen, Germany*

*^d^ Institute of Functional Interfaces (IFG), Karlsruhe Institute of Technology (KIT), Hermann-von-Helmholtz-Platz 1, 76344 Eggenstein-Leopoldshafen, Germany*

**Supplementary Information (SI)**

Fig.S1. Raw Raman spectra showing the specific Raman bands for the membrane and the Raman band
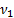
 of sulphate, which were used for analysis. Raman intensity is obtained by integrating from 1165 to 1060 cm^-1^ for the membrane (membrane signal) and from 994 to 965 cm^-1^ for sulphate (sulphate signal).

Fig.S2. Calibration data set: depth profiles recorded for multiple concentrations with no filtration operating conditions (unpressurized), u = 0.2 ms^-1^, p = 0.14 bar (unpressurized operation). SD: standard deviation

Table S3. Calibration data for each depth point z = -20 µm to z = 170 µm with Δz = 10 µm correlating Raman intensity to sulphate concentration

| Depth position (*z*) | Offset | Slope | R^2^ (COD) |
| --- | --- | --- | --- |
| 170 | -1.360 | 1.717 | 0.999 |
| 160 | -1.639 | 1.714 | 0.999 |
| 150 | -1.402 | 1.678 | 0.999 |
| 140 | -2.040 | 1.706 | 0.998 |
| 130 | -2.048 | 1.687 | 0.999 |
| 120 | -2.499 | 1.696 | 0.999 |
| 110 | -2.977 | 1.698 | 0.998 |
| 100 | -3.375 | 1.693 | 0.999 |
| 90 | -3.814 | 1.683 | 0.998 |
| 80 | -4.233 | 1.660 | 0.998 |
| 70 | -5.098 | 1.644 | 0.998 |
| 60 | -5.366 | 1.598 | 0.997 |
| 50 | -6.081 | 1.558 | 0.998 |
| 40 | -6.460 | 1.484 | 0.997 |
| 30 | -6.866 | 1.372 | 0.995 |
| 20 | -6.799 | 1.213 | 0.995 |
| 10 | -6.213 | 0.994 | 0.993 |
| 0 | -4.136 | 0.651 | 0.978 |
| -10 | -4.156 | 0.538 | 0.986 |
| -20 | -3.222 | 0.418 | 0.988 |
